# Comparison of low-molecular-weight heparins in thromboprophylaxis of major orthopaedic surgery – randomized, prospective pilot study

**DOI:** 10.1515/med-2020-0213

**Published:** 2020-10-12

**Authors:** Jan Biławicz, Michał Lipa, Miroslaw Wielgos

**Affiliations:** 1st Department of Anaesthesiology and Intensive Care, Lindley St. 4, 02-005, Warsaw, Poland; 1st Department of Obstetrics and Gynecology Medical University of Warsaw, Starynkiewicz Square 1/3, 02-15, Warsaw, Poland

**Keywords:** thrombosis, embolism, enoxaparin, dalteparin

## Abstract

**Aim:**

To compare the clinical effectiveness of the two most commonly used LMWHs, dalteparin (DALT) and enoxaparin (ENOX), in thromboprophylaxis of elective total hip replacement (THR) or total knee replacement (TKR).

**Material and methods:**

To the prospective, randomized study were included 66 adult patients qualified to undergo THR or TKR (age 63 ± 12 years, 44 women). The patients were randomized to daily in-hospital subcutaneous prophylaxis with 5,000 I.U. of DALT or 40 mg of enoxaparin. Clinical and laboratory data were collected before surgery, and on 1st and 5th days after surgery.

**Results:**

Thirty-four patients were randomized to prophylaxis with ENOX and 32 with DALT. The groups did not differ significantly in age, sex, creatinine and most of the laboratory parameters. The compared groups had similar surgical parameters, but more patients in the ENOX group received red blood cell infusion (17(50%) vs 8(25%); *p* < 0.05). The Lee–White coagulation time mildly decreased in ENOX and DALT following the surgery (*p* = ns). There was a shortening of Duke’s bleeding time in DALT after the surgery and it became significantly quicker than that in ENOX on Day 5 (*p* = 0.03).

**Conclusion:**

The observed difference in Duke’s bleeding time and exceeding blood loss during the surgery on the enoxaparin demands confirmation, as it can be important information for clinical management.

## 1 Introduction

The risk of postoperative thrombosis and bleeding in orthopaedic patients differs between procedures and patients and is related to various risk factors, all of which should be assessed prior to surgery including the one associated with anticoagulants. An individual patient’s risk is highly variable and the best method of thromboprophylaxis should be judiciously chosen. Total knee replacement (TKR) and total hip replacement (THR) surgeries carry a particularly high risk for venous thromboembolism (VTE), which includes deep vein thrombosis (DVT) and pulmonary embolism (PE) [1]. About 5% of patients undergoing a major orthopaedic surgery could have symptomatic VTE without prophylaxis. The antithrombotic prophylaxis reduces the incidence of DVT and PE up to 60% [2,3]. Patients undergoing THR or TKR are recommended to receive a minimum of 10 to 14 days of thromboprophylaxis with low-molecular-weight heparins (LMWHs), a direct thrombin inhibitor suchas dabigatran, as well as factor Xa inhibitors such as apixaban, fondaparinux and rivaroxaban, low-dose unfractionated heparin, adjusted-dose vitamin K antagonist, aspirin or an intermittent pneumatic compression device [1]. Despite the growing popularity of direct oral anticoagulants (DOACs), the LMWHs remain the first choice for in-hospital prophylaxis [4].

The LMWHs present mainly the anti-Xa activity as they contain less than 30% of high-molecular-weight molecules, showing both anti-Xa and anti-IIa actions [5]. Animal studies suggested that a high value of anti-Xa to anti-IIa activity may be related to a reduced tendency to cause bleeding [6]; however, it has not yet been confirmed in clinical trials. It is suggested that in spite of diverse production technologies, the anti-Xa activity and inhibition of the haemostatic system activation of different LMWHs are very similar [7].

Perioperative care in orthopaedics remains a crucial aspect in recovery and long-term outcomes, especially in elderly patients. Dalteparin (DALT) and enoxaparin (ENOX) are the most commonly used LMWHs in thromboprophylaxis before elective orthopaedic surgeries; however, there is no clear evidence to suggest that each molecule may be more favourable than the other one. Therefore, we decided to compare the biological activity and clinical outcomes of those two drugs in patients undergoing orthopaedic surgeries to define a more favourable substance among the above-mentioned LMWHs.

## 2 Methods

The protocol of this study was approved by the Bioethics Committee of the Medical University of Warsaw (Poland) and all participating patients signed prior to the consent form. The standards applied in this study followed ICH–GCP and the Declaration of Helsinki; however, the protocol of the study had not been published on a public clinical trial website.

To the prospective, randomized study were included 66 adult patients qualified to undergo THR or TKR (age 63 ± 12 years, 44 women). The exclusion criteria included contraindication to anticoagulation such as active major bleeding, high risk of haemorrhage, recent haemorrhagic stroke, gastrointestinal ulcer, presence of malignant neoplasm, recent brain, spinal or ophthalmic surgery, known or suspected oesophageal varices, arteriovenous malformations, vascular aneurysms or major intraspinal or intracerebral vascular abnormalities; moreover, the history of immune-mediated heparin-induced thrombocytopenia within the past 100 days or in the presence of circulating antibodies, known hypersensitivity to heparin or pork products. The patients were randomized to daily in-hospital subcutaneous prophylaxis with 5,000 I.U. of DALT (Fragmin, Pfizer Europe MA EEIG, Belgium) or 40 mg of ENOX (Clexane, Sanofi-Aventis, France). The initial dosage of LMWH was given 12 h before the planned surgery. Subsequent doses were administered once daily with the restriction of the postoperative dose at least 12 h after surgery. Immediately before surgery, all patients received analgesia with epidural blockade using 0.5% marcain spinal heavy (Aspen Pharma, South Africa) injected subarachnoid with a 26G needle (Atraucan, B. Braun Melsungen AG, Germany). Clinical and laboratory data were collected before surgery, on the 1st and 5th days after surgery. The clinical assessment included the general status, heart rate, blood pressure and signs of bleeding. Blood samples were collected for blood cell count, sodium, potassium, creatinine, prothrombin time (PT), activated partial thromboplastin time (APTT), thrombin time (TT), fibrinogen concentration, Duke’s bleeding time and Lee–White coagulation time. The blood loss during the surgery was assessed by the performing surgeon, the anaesthetist and the amount of transfused fluids and red blood cells.

## 3 Statistics

The continuous data were presented as mean followed by standard deviation or median with range and Student’s *t*-test or Mann–Whitney’s test was used for the assessment of differences between groups, respectively. To compare the discrete variables, the chi square test with Yates’ correction and Fisher’s exact test were used. Pearson’s correlation coefficient was calculated to evaluate the correlation between variables. All tests were two sided and a value of *p* < 0.05 was considered statistically significant. Calculations were performed using STATISTICA software.

## 4 Results

Among 34 patients randomized to prophylaxis with ENOX, 17 had TKR and 17 THR. The remaining 32 patients received DALT and the TKR was performed in 16 cases, while the remaining 16 underwent THR. The groups did not differ significantly in age, sex, creatinine and most laboratory parameters (Table 1). The only difference was noticed in APTT, which was slightly longer in DALT (32.5 ± 4.0 vs 30.4 ± 3.3; *p* = 0.02).

**Table 1:** Patients’ basic characteristic before surgery

Variable	All patients *n* = 66	Enoxaparin *n* = 34	Dalteparin *n* = 32	*p*
Age [years]	63 ± 12	64 ± 13	62 ± 11	0.82
Sex [M/W]	22/44	14/20	8/24	0.16
Body mass [kg]	75 ± 15	76 ± 12	75 ± 18	0.94
Heart rate [1/min]	75 ± 10	74 ± 9	77 ± 11	0.18
Mean arterial pressure [mmHg]	101 ± 10	101 ± 9	100 ± 12	0.91
Red blood count [*10^6^/µL]	4.6 ± 0.4	4.6 ± 0.4	4.6 ± 0.4	0.94
Haematocrit [%]	41 ± 3.8	42 ± 4	41 ± 4	0.54
Haemoglobin [g/L]	139 ± 12	140 ± 12	138 ± 13	0.89
Platelet [×10^3^/µL]	250 ± 61	241 ± 59	259 ± 63	0.24
Creatinine [mg/dL]	0.83 ± 0.20	0.85 ± 0.18	0.81 ± 0.22	0.86
PT [s]	12.9 ± 1.9	12.7 ± 1.7	13.1 ± 2.1	0.45
International normalized ratio (INR)	1.05 ± 0.11	1.06 ± 0.10	1.05 ± 0.11	0.58
APTT [s]	31.4 ± 3.8	30.4 ± 3.3	32.5 ± 4.0	0.02
TT [s]	14.6 ± 1.9	14.6 ± 1.6	14.7 ± 2.2	0.78
Fibrinogen [g/L]	3.8 ± 0.8	3.8 ± 0.7	3.8 ± 0.9	0.92
Duke’s bleeding time [s]	145 ± 49	145 ± 44	145 ± 55	0.99
Lee–White coagulation time [s]	402 ± 111	394 ± 118	411 ± 104	0.54

Both groups had similar surgical parameters including surgical environment, bleeding during the procedure and achieved haemostasis (Table 2); however, more patients in ENOX received red blood cell infusion (17(50%) vs 8(25%); *p* < 0.05). There was no thrombotic event (DVT nor PE) nor major bleeding during the 3-month follow-up.

**Table 2:** Surgery-related characteristics: procedure type, blood loss and transfused fluids

Variable	All patients *n* = 66	Enoxaparin *n* = 34	Dalteparin *n* = 32	*p*
Procedure [TKR/THR]	33/33	17/17	16/16	—
Bleeding according to the anaesthesiologist [minimal/moderate/severe]	36/28/2	17/15/2	19/13/0	0.54
Bleeding according to the surgeon [minimal/moderate/severe]	34/31/1	18/15/1	16/16/0	0.74
Surgery environment [good/moderate/poor]	45/10/11	21/5/8	24/5/3	0.30
Haemostasis [good/moderate/poor]	55/11/0	27/7/0	28/4/0	0.58
Fluids in total [mL]	3,000 (1,500–4,000)	3,000 (1,500–4,000)	3,000 (2,000–4,000)	0.72
Crystalloids [mL]	2,500 (1,000–3,500)	2,500 (1,000–3,500)	2,250 (1,500–3,000)	0.64
Colloids [mL]	500 (500–2,500)	500 (500–2,500)	500 (500–1,000)	0.32
Red blood cells [mL]	0 (0–1,040)	150 (0–600)	0 (0–1,040)	0.04
Number of patients receiving RBCs *n*(%)	25 (38%)	17 (50%)	8 (25%)	<0.05

TKR, total knee replacement; THR, total hip replacement; RBC, red blood cell.

**Figure 1:**
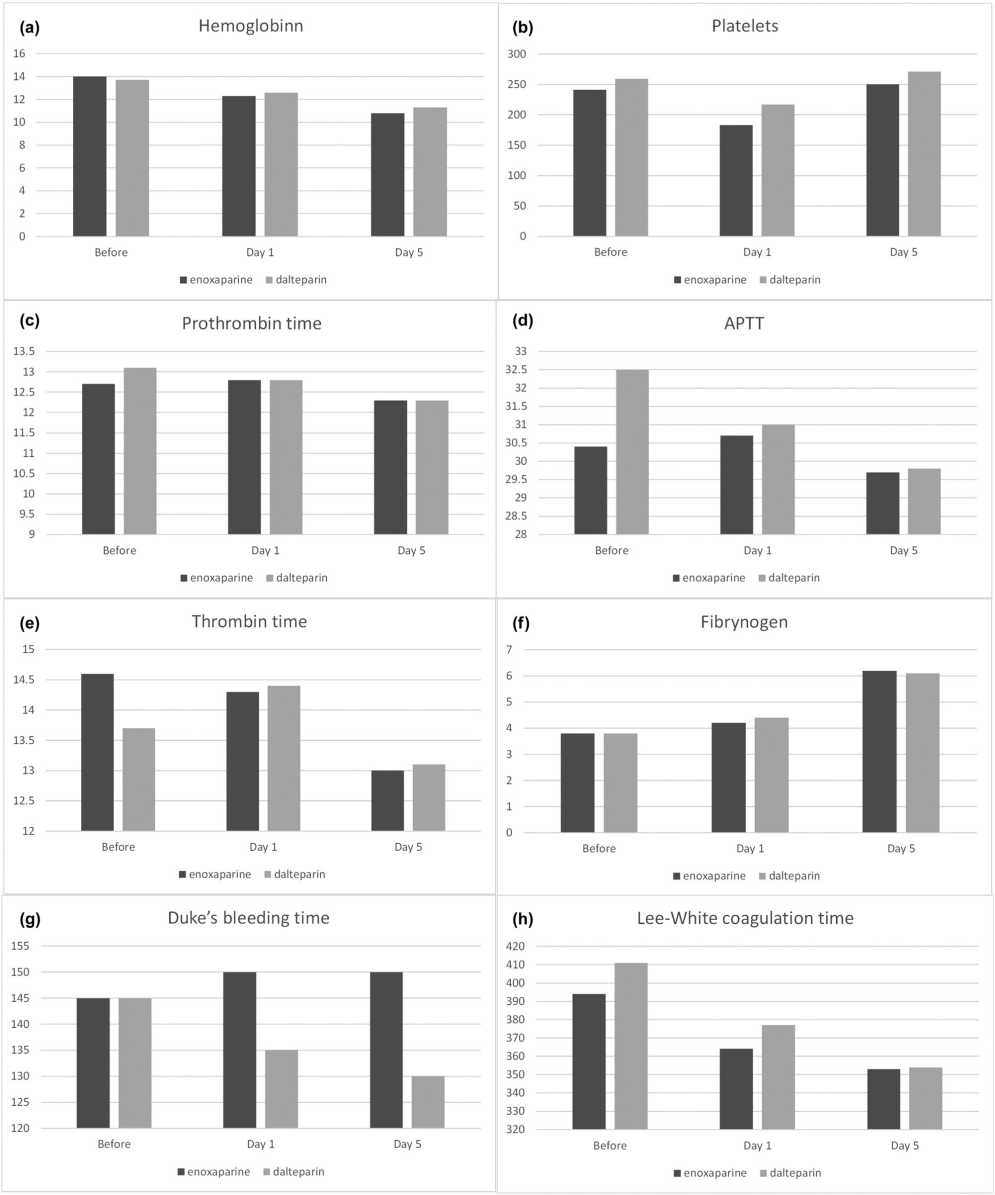
Comparison of the blood results among the study group.

On the 1st and 5th days, a similar and significant decline in haemoglobin concentration was noticed in both groups (ENOX 140 ± 12, 123 ± 14 and 108 ± 16 g/L; *p* < 0.0001; DALT 138 ± 13, 126 ± 13 and 113 ± 14 g/L; *p* < 0.0001) (Figure 1a). Platelet count decreased on the 1st day but rose back to a similar number as before surgery on the 5th day in both groups (ENOX 241 ± 59, 184 ± 49 and 250 ± 62; *p* < 0.0001; DALT 259 ± 63, 217 ± 52 and 271 ± 77; *p* < 0.0001) (Figure 1b). However, this reduction was more pronounced in ENOX leading to a significant difference between the groups (184 ± 49 vs 217 ± 52; *p* < 0.01) on the 1st day. PT shortened after the surgery in both groups, but significantly only in ENOX (12.7 ± 1.7, 12.8 ± 1.3 and 12.3 ± 0.8 s; *p* = 0.01) (Figure 1c). After the surgery, APPT declined only in DALT (32.5 ± 4.0, 31.0 ± 3.9 and 29.8 ± 3.9 s; *p* < 0.0001) and in the end it did not differ from ENOX in the follow-up (Figure 1d). In both groups, TT reduced (ENOX 14.6 ± 1.6, 14.3 ± 1.5 and 13.0 ± 1.4 s; *p* < 0.0001; DALT 14.7 ± 2.2, 14.4 ± 0.9 and 13.1 ± 1.6 s; *p* < 0.0001) (Figure 1e), while the fibrinogen concentration rose (ENOX 3.8 ± 0.7, 4.2 ± 1.0 and 6.2 ± 0.9; *p* < 0.0001; DALT 3.8 ± 0.9, 4.4 ± 0.8 and 6.1 ± 0.9; *p* < 0.0001) post TKR and THR (Figure 1f). Following the surgery, Lee–White coagulation time mildly decreased in ENOX and DALT, but the fall did not achieve statistical significance (Figure 1g). Interestingly, there was a shortening of Duke’s bleeding time in DALT after the surgery and in the end, it was significantly shorter than in ENOX on the 5th day (130 ± 36 vs 150 ± 38 s; *p* = 0.03) (Figure 1h).

Duke’s bleeding time in ENOX, but not in DALT, correlated with age (*r* = 0.38 *p* = 0.03) and reversely with the total amount of fluids and crystalloids transfused during the procedure (*r* = (−0.39) *p* = 0.02 and *r* = (−0.39) *p* = 0.02, respectively).

## 5 Discussion

LMWHs remain a gold standard in the prophylaxis of VTE in a major orthopaedic surgery, though they have not shown themselves to be superior to any of the other chemoprophylactic agents. As thrombotic events are one of the most severe complications in perioperative period, we shall pay special attention to the thromboprophylaxis standards. It seems that the increased risk of bleeding remains a less significant aspect in clinical practice, when compared to the severe, thrombotic complications. Nevertheless, LMWHs stay widely used in thromboprophylaxis after major orthopaedic surgeries, notwithstanding a growing usage of DOACs. In comparison of thromboprophylaxis following arthroplasty used by Australian orthopaedic surgeons reported in 2012 and 2017, most respondents stated using anticoagulants (88.9% and 98.6%, respectively); of these, the most preferred injectable agents alone, and generally ENOX [4].

To our knowledge, this is one of just the few studies investigating the clinical usefulness of the two LMWHs – ENOX and DALT and the only one undertaken as prospective and randomized. According to the available literature, most of the studies are retrospective and focus mainly on the frequency of venous thromboembolism, based on the clinical data. This study not only investigates this matter, but also provides a comprehensive analysis of the *in vivo* performance of the two LMWHs, based on various laboratory parameters combined with clinical outcomes. The above-mentioned, retrospective studies revealed that there is no significant difference in the frequency of VTE in acute trauma patients treated with ENOX or DALT and both substances may be considered as equivalents [8,9].

In our study, both groups had similar surgical parameters, but more patients in ENOX received red blood cell infusion. We also noticed a shorter Duke’s bleeding time after the surgery in patients receiving DALT than ENOX (130 ± 36 vs 150 ± 38 s; *p* = 0.03). The above-mentioned observations reflect the pharmacokinetic and pharmacodynamic differences between the LMWHs, which are well absorbed from subcutaneous administration, but the bioavailability of anti-Xa activity varies from about 87% for DALT, about 91% for ENOX, up to 98% for nadroparin [10]. They are metabolized in the liver to inactive fragments, while the rest is eliminated by the kidneys [5]. In a comparative study between ENOX 20 mg and 40 mg, DALT 2,500 IU, and nadroparin 7,500 IU injected subcutaneously, the average apparent total body clearance of ENOX was 15.6 mL/min. This was substantially lower than those of DALT (33 mL/min) and nadroparin (21.4 mL/min) [10]. Therefore, DALT is cleared from the body more rapidly than nadroparin and ENOX. Importantly, for ENOX 20 mg and 40 mg, urinary excretion represents 6.4% and 8.7% of the injected dose, which differs from those of nadroparin (3.9%) and DALT (3.4%) [10]. ENOX has a longer apparent half-life (mean 4.1 h) in the anti-Xa assay than DALT (mean 2.8 h) and nadroparin (mean 3.7 h), which is a reflection of their respective clearance values [10]. LMWHs’ anti-Xa clearance decreases with the degree of renal function [7].

The retrospective, observational, cross-sectional, cohort analysis of 1,13,936 patients after a major orthopaedic surgery revealed a similar rate of VTE in the group receiving thromboprophylaxis with DALT (2.1%) and the group with ENOX (2.3%) as the prevalence of major bleeding (1.1% vs 1.5%, respectively) [11]. Our prospective and randomized observation also found no difference in the major bleeding, but suggested a more intensive blood loss during the surgery.

## 6 Study limitations

This is a small, single centre pilot study, although it is prospective, randomized and allowed to indicate the potential area of interest and further investigation.

## 7 Conclusion

ENOX and DALT seem to have a similar impact on the laboratory parameters of coagulation. Nevertheless, the observed difference in Duke’s bleeding time and exceeding blood loss during the surgery on enoxaparin demands confirmation, as it can be important information for clinical management.
